# Supplementary material for: Information sharing and channel encroachment in biomass supply chains
Source: PLoS One. 2024 Sep 20;19(9):e0310539. doi: 10.1371/journal.pone.0310539 (PMC11414914; doi:10.1371/journal.pone.0310539)
Supplement: S1 File — (PDF) [file pone.0310539.s001.pdf]

Appendix 1 Derivation process

Proofs of Scenarios NN, NM, EN and EM

Using the equality $E(\pi_F) = (w_1 - c_1)d_1$, we have $\frac{\partial^2 E(\pi_F)}{\partial w_1^2} = -2 < 0$, which shows that $E(\pi_F)$ is concave in w_1. From $\frac{\partial E(\pi_F)}{\partial w_1} = 0$, we get the reaction function $w_1 = A + c_1 - p_1$. Since the farmer does not know the demand information, he will therefore set a fixed wholesale price $w_1 = a_0 + c_1 - p_1$. Substituting $w_1 = a_0 + c_1 - p_1$ into the equality $E(\pi_M) = (p_1 - w_1 - c_2)d_1$, we have $\frac{\partial^2 E(\pi_M)}{\partial p_1^2} = -4 < 0$, which shows that $E(\pi_M)$ is concave in p_1. From $\frac{\partial E(\pi_M)}{\partial p_1} = 0$, we have $p_1 = \frac{1}{4}(3a_0 + c_1 + c_2)$. Substituting $p_1 = \frac{1}{4}(3a_0 + c_1 + c_2)$ into the aforementioned $w_1 = a_0 + c_1 - p_1$, we obtain the suitable outcomes for Scenario NN.

Using a similar approach, we can obtain the equilibrium result for Scenarios NM, EN and EM, and the proof is omitted here.

Proofs of Scenarios NF, NFM, EF and EFM

Using the equality $E(\pi_F) = (w_1 - c_1)d_1$, we have $\frac{\partial^2 E(\pi_F)}{\partial w_1^2} = -2 < 0$, which shows that $E(\pi_F)$ is concave in w_1. From $\frac{\partial E(\pi_F)}{\partial w_1} = 0$, we get the reaction function $w_1 = A + c_1 - p_1$. Since the middleman does not know the demand information, he will set his retail price based on the expectation of the wholesale price. Thus, substituting $w_1 = a_0 + c_1 - p_1$ into the equality $E(\pi_M) = (p_1 - w_1 - c_2)d_1$, we have $\frac{\partial^2 E(\pi_M)}{\partial p_1^2} = -4 < 0$, which shows that $E(\pi_M)$ is concave in p_1. From $\frac{\partial E(\pi_M)}{\partial p_1} = 0$, we have $p_1 = \frac{1}{4}(3a_0 + c_1 + c_2)$. Substituting $p_1 = \frac{1}{4}(3a_0 + c_1 + c_2)$ into the aforementioned $w_1 = A + c_1 - p_1$, we obtain the suitable outcomes for Scenario NF.

Using a similar approach, we can obtain the equilibrium result for Scenarios NFM, EF and EFM, and the proof is omitted here.

Proofs of Propositions

Proposition 1. By comparing with the equilibrium results, we have: $\mathrm{w}_1^{\mathrm{NM}} - \mathrm{w}_1^{\mathrm{NN}} = \frac{a_0 - A}{2}$; $\mathrm{w}_1^{\mathrm{NN}} - \mathrm{w}_1^{\mathrm{NFM}} = \frac{a_0 - A}{4}$; $\mathrm{w}_1^{\mathrm{NFM}} - \mathrm{w}_1^{\mathrm{NF}} = \frac{3(a_0 - A)}{4}$. Therefore, when $\mathrm{A} > \mathrm{a}_0$, $\mathrm{w}_1^{\mathrm{NM}} < \mathrm{w}_1^{\mathrm{NN}} < \mathrm{w}_1^{\mathrm{NFM}} < \mathrm{w}_1^{\mathrm{NF}}$. In a similar way, we can get the rest of Proposition 1.

Propositions 2-5. Using a similar logic as in Proposition 1, we obtain Propositions 2-5, and the proof is omitted here.
