# Supplementary material for: Information sharing and channel encroachment in biomass supply chains
Source: PLoS One. 2024 Sep 20;19(9):e0310539. doi: 10.1371/journal.pone.0310539 (PMC11414914; doi:10.1371/journal.pone.0310539)
Supplement: Attachment — Submitted filename: Response to Reviewers.docx [file pone.0310539.s002.docx]

**Authors response to** **Academic Editor's and Reviewers' Comments on** **PONE-D-24-05811.**

On behalf of my co-authors, we really appreciate for the opportunity to revise our manuscript. We also would like to thank the academic editor and reviewers for their comments and suggestions to improve the paper, which have been incorporated in the present revised version. Our response to each one of the academic editor's and reviewer’s reports is provided below.

**Response to Academic Editor’s Comments**

**Answer:** Thanks for your valuable comments and suggestions. We will answer your comments one by one.

**Comment (1)**: Please ensure that your manuscript meets PLOS ONE's style requirements, including those for file naming. The PLOS ONE style templates can be found at

https://journals.plos.org/plosone/s/file?id=wjVg/PLOSOne_formatting_sample_main_body.pdf and

https://journals.plos.org/plosone/s/file?id=ba62/PLOSOne_formatting_sample_title_authors_affiliations.pdf.

**Answer:** We feel great thanks for your professional review work on our article. As you are concerned, there are several problems that need to be addressed. According to your nice suggestions, we have made partial corrections to our previous draft, the detailed corrections are listed below.

(i) We have changed the font size and typeface of Level 1 headings, Level 2 headings, and Level 3 headings as required.

(ii) Figure captions in the text have been formatted in bold type as requested. Formulas in the text have been numbered according to the requirements of the standard text format.
 (iii) The graphic files have been uploaded to the Prepress Check Analysis and Conversion Engine (PACE) to generate .tiff files, and then I inserted the generated .tiff files into my article.

**Comment (2)**: Please note that PLOS ONE has specific guidelines on code sharing for submissions in which author-generated code underpins the findings in the manuscript. In these cases, all author-generated code must be made available without restrictions upon publication of the work. Please review our guidelines at https://journals.plos.org/plosone/s/materials-and-software-sharing#loc-sharing-code and ensure that your code is shared in a way that follows best practice and facilitates reproducibility and reuse.

**Answer:** Thank you for the constructive suggestions.

(i)The formula calculations in the article are hand-calculated and the derivation process is as follows：

**Proofs of Scenarios NN, NM, EN and EM**

Using the equality $E\left( \pi_{F} \right)=\left( w_{1}-c_{1} \right)d_{1}$, we have $\frac{\partial^{2}E\left( \pi_{F} \right)}{\partial w_{1}^{2}}=-2<0$, which shows that $E\left( \pi_{F} \right)$ is concave in $w_{1}$. From $\frac{\partial E\left( \pi_{F} \right)}{\partial w_{1}}=0$, we get the reaction function $w_{1}=A+c_{1}-p_{1}$. Since the farmer does not know the demand information, he will therefore set a fixed wholesale price $w_{1}=a_{0}+c_{1}-p_{1}$. Substituting $w_{1}=a_{0}+c_{1}-p_{1}$ into the equality $E\left( \pi_{M} \right)=\left( p_{1}-w_{1}-c_{2} \right)d_{1}$, we have $\frac{\partial^{2}E\left( \pi_{M} \right)}{\partial p_{1}^{2}}=-4<0$, which shows that $E\left( \pi_{M} \right)$ is concave in $p_{1}$. From $\frac{\partial E\left( \pi_{M} \right)}{\partial p_{1}}=0$, we have $p_{1}=\frac{1}{4}(3a_{0}+c_{1}+c_{2})$. Substituting $p_{1}=\frac{1}{4}(3a_{0}+c_{1}+c_{2})$ into the aforementioned $w_{1}=a_{0}+c_{1}-p_{1}$, we obtain the suitable outcomes for Scenario NN.

Using a similar approach, we can obtain the equilibrium result for Scenarios NM, EN and EM, and the proof is omitted here.

**Proofs of Scenarios NF, NFM, EF and EFM**

Using the equality $E\left( \pi_{F} \right)=\left( w_{1}-c_{1} \right)d_{1}$, we have $\frac{\partial^{2}E\left( \pi_{F} \right)}{\partial w_{1}^{2}}=-2<0$, which shows that $E\left( \pi_{F} \right)$ is concave in $w_{1}$. From $\frac{\partial E\left( \pi_{F} \right)}{\partial w_{1}}=0$, we get the reaction function $w_{1}=A+c_{1}-p_{1}$. Since the middleman does not know the demand information, he will set his retail price based on the expectation of the wholesale price. Thus, substituting $w_{1}=a_{0}+c_{1}-p_{1}$ into the equality $E\left( \pi_{M} \right)=\left( p_{1}-w_{1}-c_{2} \right)d_{1}$, we have $\frac{\partial^{2}E\left( \pi_{M} \right)}{\partial p_{1}^{2}}=-4<0$, which shows that $E\left( \pi_{M} \right)$ is concave in $p_{1}$. From $\frac{\partial E\left( \pi_{M} \right)}{\partial p_{1}}=0$, we have $p_{1}=\frac{1}{4}(3a_{0}+c_{1}+c_{2})$. Substituting $p_{1}=\frac{1}{4}(3a_{0}+c_{1}+c_{2})$ into the aforementioned $w_{1}=A+c_{1}-p_{1}$, we obtain the suitable outcomes for Scenario NF.

Using a similar approach, we can obtain the equilibrium result for Scenarios NFM, EF and EFM, and the proof is omitted here.

**Proofs of Propositions**

**Proposition 1.** By comparing with the equilibrium results, we have: $w_{1}^{\mathrm{NM}}-w_{1}^{\mathrm{NN}}=\frac{a_{0}-A}{2}$; $w_{1}^{\mathrm{NN}}-w_{1}^{\mathrm{NFM}}=\frac{a_{0}-A}{4}$; $w_{1}^{\mathrm{NFM}}-w_{1}^{\mathrm{NF}}=\frac{3(a_{0}-A)}{4}$. Therefore, when $A>a_{0}$, $w_{1}^{\mathrm{NM}}<w_{1}^{\mathrm{NN}}<w_{1}^{\mathrm{NFM}}<w_{1}^{\mathrm{NF}}$. In a similar way, we can get the rest of Proposition 1.

**Propositions 2-5.** Using a similar logic as in Proposition 1, we obtain Propositions 2-5, and the proof is omitted here.

(ii) The figure in the article were generated using Mathematica software. For example，similar to existing studies Wang et al. [50] and Liu et al.[56],$\mathrm{we} \mathrm{set}a_{0}=15\text{;}c_{1}=2;c_{2}=1;c_{3}=0.8;c_{0}=2.5;v=1.$Among them, the left figure is $b=0.3$, and the right is $b=0.6$, and brought into the equilibrium solution. The following code can be run in Mathematica software to obtain Figure 4

Plot[{E[ \!\(\*SubsuperscriptBox[\(\[Pi]\), \(P\), \

\(\(EN\)\(*\)\)]\) ],

E[\!\(\*SubsuperscriptBox[\(\[Pi]\), \(P\), \

\(\(EF\)\(*\)\)]\)],

E[\!\(\*SubsuperscriptBox[\(\[Pi]\), \(P\), \

\(\(EM\)\(*\)\)]\)],

E[\!\(\*SubsuperscriptBox[\(\[Pi]\), \(P\), \

\(\(EFM\)\(*\)\)]\)]}, {t, 0, 1},

PlotStyle -> {{Red, Dashed}, {Red}, {Blue}, {Orange}},

PlotLegends ->

Placed[LineLegend[

Style[TraditionalForm[#],

7] & /@ {"E[ \!\(\*SubsuperscriptBox[\(\[Pi]\), \(P\), \

\(\(EN\)\(*\)\)]\) ]",

"E[\!\(\*SubsuperscriptBox[\(\[Pi]\), \(P\), \

\(\(EF\)\(*\)\)]\)]",

"E[\!\(\*SubsuperscriptBox[\(\[Pi]\), \(P\), \

\(\(EM\)\(*\)\)]\)]",

"E[\!\(\*SubsuperscriptBox[\(\[Pi]\), \(P\), \

\(\(EFM\)\(*\)\)]\)]"}, LegendLayout -> {"Row", 2}], {0.4, 0.7}],

Frame -> True, PlotRangePadding -> None,

FrameLabel -> {"t", "E[\!\(\*SubscriptBox[\(\[Pi]\), \(P\)]\)]"},

LabelStyle -> Directive[15]]

**Comment (3)**: PLOS requires an ORCID iD for the corresponding author in Editorial Manager on papers submitted after December 6th, 2016. Please ensure that you have an ORCID iD and that it is validated in Editorial Manager. To do this, go to ‘Update my Information’ (in the upper left-hand corner of the main menu), and click on the Fetch/Validate link next to the ORCID field. This will take you to the ORCID site and allow you to create a new iD or authenticate a pre-existing iD in Editorial Manager. Please see the following video for instructions on linking an ORCID iD to your Editorial Manager account: <https://www.youtube.com/watch?v=_xcclfuvtxQ>".

**Answer:** Thank you for your comments. We have linked ORCID iD: 0000-0001-5009-6416 to the Edit Manager account.

**Comment (4)**: Thank you for stating the following in the Acknowledgments Section of your manuscript:

[This study was supported by Henan province agricultural science and technology research project (Grant No: 192102110205).]

We note that you have provided funding information that is not currently declared in your Funding Statement. However, funding information should not appear in the Acknowledgments section or other areas of your manuscript. We will only publish funding information present in the Funding Statement section of the online submission form.

Please remove any funding-related text from the manuscript and let us know how you would like to update your Funding Statement. Currently, your Funding Statement reads as follows:

[The author(s) received no specific funding for this work.]

Please include your amended statements within your cover letter; we will change the online submission form on your behalf.

**Answer:** Thank you for your valuable comments. We have removed any funding related text from the manuscript. The "Funding Statement" of the manuscript reads as follows: [The author(s) received no specific funding for this work.

**Comment (5)**: We note that your Data Availability Statement is currently as follows: [All relevant data are within the manuscript and its Supporting Information files.]

Please confirm at this time whether or not your submission contains all raw data required to replicate the results of your study. Authors must share the “minimal data set” for their submission. PLOS defines the minimal data set to consist of the data required to replicate all study findings reported in the article, as well as related metadata and methods (<https://journals.plos.org/plosone/s/data-availability#loc-minimal-data-set-definition>).

**Answer:** Thank you for your valuable advice. After our review, the submitted manuscript contains all the original data needed to reproduce the findings of the study. Details are as follows:

(i) The data cited in the introduction of this paper are all from existing research articles and websites, and are reflected in the references.

(ii) No specific data was used in the calculation of balance results in this paper. Please refer to the appendix for the proof process. Therefore, there is no data such as mean, standard deviation, and other measurements.

(iii) In this paper, similar to the drawing part of Wang et al. [50] and Liu et al. [56], specific parameters (specified parameters have been indicated in the paper) are set by numerical simulation and input into the equilibrium result, which can be obtained by running the code in the mathematical software Mathematica.

**Comment (6)**: Please review your reference list to ensure that it is complete and correct. If you have cited papers that have been retracted, please include the rationale for doing so in the manuscript text, or remove these references and replace them with relevant current references. Any changes to the reference list should be mentioned in the rebuttal letter that accompanies your revised manuscript. If you need to cite a retracted article, indicate the article’s retracted status in the References list and also include a citation and full reference for the retraction notice.

**Answer:** Thank you for your significant reminder. As per the stipulations, we conducted a thorough review of the references in the article, made revisions to certain references, and did not identify any articles in a retracted status.

**Additional Editor Comments:** The abstract section should be revised, and the research gap should be also addressed in the first section

**Answer:** Based on your valuable comments, we have revised the abstract and the first section as follows.

(i) We have modified the presentation of some of the conclusions in the abstract:

“When the power plant shares its information only with the middleman, it can maximize its profits, which, however, may not be conducive to the long-term stability of the supply chain. Furthermore, surprisingly, in the absence of channel encroachment, the power plant sharing information with upstream members may harm their profits.”

(ii) In order to highlight the research gaps between this study and previous literature, we have described them in points:

“This study makes several contributions to the literature as follows. Firstly, it focuses on the information sharing strategy of a biomass supply chain consisting of a power plant, a middleman, and farmers. This supply chain structure is common in biomass energy supply but lacks related research on information sharing strategies. Secondly, we derive pricing equilibrium strategies for different information sharing scenarios, using whether farmers choose the encroachment channel as an entry point. Thirdly, we further compared the expected profits of biomass supply chain members in different channel information scenarios.”

**Response to Reviewer: 1’s Comments**

**Comment:** This paper investigates the interactions between the information sharing strategy and channel encroachment in the straw power supply chain. I Think this paper is interesting, but several problems still need to be addressed.

**Answer:** Thanks for your valuable comments and suggestions. We will answer your comments one by one.

**Comment (1):** The first part introduces the background and motivation of the research, and the general idea is good. It would be more helpful to offer more examples, especially information sharing.

**Answer:** We have added three case studies on biomass information sharing and policy-led biomass information sharing platform building in Part I, aiming to enrich the real-world basis of this study. For your convenience, we repeat it here:

(i) In the comprehensive utilization of straw in Jiaxian County, Henan Province, the government and enterprises take the lead in building a straw collection and storage information platform, so that the utilization rate of straw in the county has reached more than 80%.

(ii) In order to promote crop straw storage and comprehensive utilization, Guiyang Municipal Bureau of Agriculture and Rural Development organized the city's straw storage and utilization of the main demand for information and to be announced on the official website.

(iii) In 2022, the National Development and Reform Commission (NDRC)'s guidance for green and low-carbon energy transformation also explicitly stated the need to build an energy basic information platform and integrate energy supply chain information.

**Comment (2):** The second part reviews the literature of the three related fields. Authors are also advised to increase citations from the PLOS ONE journal. It is suggested to list the relevant literature, contributions, as well as the innovation of this paper in the form of a table.

**Answer:** We feel great thanks for your professional review work on our article. As you are concerned, there are several problems that need to be addressed.

(i) The comparison of our paper with the extant literature is shown in Table 1.

**Table 1. Comparison with the literature.**

|  | **Biomass supply chain** | | | **Channel** | | | | **Information** | | | | | | | |  |
| --- | --- | --- | --- | --- | --- | --- | --- | --- | --- | --- | --- | --- | --- | --- | --- | --- |
|  | Behavioral decision | Logistics network | Policy and risk control | | Encroachment | | Non-encroachment | | Symmetric | | Asymmetric | | Shared | | Unshared | |
| [25]  [26]  [27]  [28]  [29]  [30]  [31]  [20]  [32]  [33]  [34, 46, 47, 50, 52]  [35-38]  [39]  [40]  [41, 44]  [42, 43]  [48, 51]  This paper |  | ✓ | ✓ | |  | |  | |  | |  | |  | |  | |
|  | ✓ |  | ✓ |  | |  | |  | |  | |  | |  | |  |
|  | ✓ |  | ✓ | ✓ | | ✓ | | ✓ | |  | |  | |  | |  |
|  |  |  | ✓ |  | |  | |  | |  | |  | |  | |  |
|  |  | ✓ |  |  | |  | |  | |  | |  | |  | |  |
|  | ✓ |  |  |  | |  | |  | | ✓ | |  | |  | |  |
|  |  | ✓ |  |  | |  | |  | |  | |  | |  | |  |
|  | ✓ |  | ✓ |  | |  | |  | | ✓ | | ✓ | | ✓ | |  |
|  | ✓ |  | ✓ |  | | ✓ | | ✓ | |  | |  | |  | |  |
|  | ✓ |  |  | ✓ | |  | | ✓ | |  | |  | |  | |  |
|  |  |  |  | ✓ | | ✓ | |  | | ✓ | | ✓ | | ✓ | |  |
|  |  | ✓ |  |  | |  | |  | |  | |  | |  | |  |
|  | ✓ |  | ✓ |  | |  | |  | |  | |  | |  | |  |
|  | ✓ |  |  |  | | ✓ | |  | | ✓ | |  | |  | |  |
|  | ✓ |  |  | ✓ | |  | |  | |  | |  | |  | |  |
|  | ✓ |  | ✓ | ✓ | |  | |  | |  | |  | |  | |  |
|  |  |  |  |  | |  | |  | | ✓ | | ✓ | | ✓ | |  |
|  | ✓ |  |  | ✓ | | ✓ | |  | | ✓ | | ✓ | | ✓ | |  |

(ii) In discussing the relationship between information sharing and supply chains, we cite two research articles from PLOS ONE:

47.Liu M, Zhai L, Gan H. An evolutionary analysis of supply chain collaborative information sharing based on prospect theory. PloS one. 2024;19(3):e0298355. doi: 10.1371/journal.pone.0298355. PubMed PMID: MEDLINE:38489344.

48.de Frutos EH, Trapero JR, Ramos F. A literature review on operational decisions applied to collaborative supply chains. Plos One. 2020;15(3). doi: 10.1371/journal.pone.0230152. PubMed PMID: WOS:000535290100025.

**Comment (3):** In Section 3, I recommend adding the descriptions to explain why this decision sequence for the game is adopted.

**Answer:** Thank you for your constructive comments. In Section 3, we add a note to explain why this order of game decisions is used.

**“**The biomass power supply chain is essentially a supply chain primarily driven by production (electricity generation). The invasion decision is assumed to be made before the sharing strategy because, in general, the power plant holds a leadership position in the sup-ply chain, and after observing the upstream structure, the power plant decides whether to share information with upstream partners. Additionally, similar to Wei et al.[57], this chapter sets the corresponding pricing decision sequence.”

**Comment (4):** There are some areas of expression that need to be improved. In the first line of the second paragraph of part 3, I don't understand what function is the 'existing function' here? Where does it come from? Why is it used? What's the difference between this pre-existing function in the original literature and in my current paper?

**Answer:** Based on your comments, we have modified the formulation of the demand function. The demand function for a biomass power plant is portrayed based on a list of existing research literature:

Similar to Yan et al.[54] and Li et al. [55], the biomass plant demand is assumed to be a downward sloping linear curve of the following form .When farmers do not invade, the demand function is $d_{1}=a-p_{1}$. When farmers invade, the demand functions are $d_{1}=a-p_{1}+bp_{2}$ and $d_{2}=a-p_{2}+bp_{1}$. Here, $d_{i}$ and $p_{i}$are the demand quantity and unit price for selling biomass to the power plant in the channel $i$ (where $i=1$ and $2$ represent the middleman and farmer invasion channels, respectively), and a represents the potential demand. The parameter $b(1>b>0)$ indicates the intensity of competition between the two channels.

**Comment (5):** The paper should explain more about the mechanism behind the conclusions. Specifically, the theoretical analysis is simple and insufficient, and the reason behind some key results is not clear. For example, Corollaries 4 shows that sharing information of biomass power plants will lead to vertical competition of raw material supply channels. I would like to see a more specific description after the 'vertical competition', for example, the price war caused by vertical competition leads to the reduction of profits of biomass power plants? Other analyses have the same problem.

**Answer:** According to your nice suggestions, we have made extensive corrections to our previous draft, the detailed corrections are listed below.

(i) In Proposition 1, we provide a detailed discussion of the mechanisms behind unit price adjustments by intermediaries: “As for the unit selling price $p_{1}$ for the middlemen, it is noteworthy that regardless of farmer invasion, when demand information is exclusively held by the middlemen, the unit price $p_{1}$ actually decreases. This is because when demand information forecasts high values（$A{>a}_{0}$）, the middlemen are more willing to lower unit prices to gain more market share and maximize their profits.”

(ii) In Proposition 2, We explain the reasons behind the fact that exclusive access to information by middlemen reduces farmers' profits: “This is because when the middleman has demand information, they are likely to set a more reasonable unit selling price $p_{1}$. In this case, farmers are forced to set a wholesale price lower than $p_{1}$to get more sales.”

(iii) In Proposition 4, we specifically discuss the consequences of vertical competition:“When power plants share demand information with both upstream participants, the vertical competition between farmers and middlemen intensifies, which not only affects the stability of supply but also exposes biomass power plants to cost pressures arising from upstream supply competition, ultimately leading to a decline in their profits.”

(iv) In Proposition 5, we add a specific statement about the effect of channel competition intensity on power plant profits:“Both parties may adopt aggressive strategies in their competition for market share, potentially leading to a price war that weakens the bargaining power of biomass power plants and subsequently results in a decline in profits.”

(v) In 6.1 Major findings, we have supported our findings with existing research literature: “This finding is concordant with the conclusions drawn from existing studies, which indicate that in reality, farmers, either spontaneously or under government guidance, have formed various formal organizations and established direct channels for selling biomass to power plants [62].”

**Comment (6):** I also recommend adding several management insights in Sections 4 and 5.

**Answer:** Thanks for your valuable comments and suggestions. We have enriched the management insights in Sections 4 and 5 of the article.

(i) In Proposition 3, we suggest that middlemen should strengthen cooperation with biomass power plants and actively seek more accurate demand information in order to take advantage of the information gap so that they can obtain more benefits.

(ii) In Proposition 4,we suggests that in a single-channel supply chain structure, power plants should prioritize sharing demand information with the middleman.

(iii) In Proposition 6, we suggest through real-life examples that farmers should broaden their supply channels: “This is consistent with reality, as in some countries, an increasing number of farmers are forming associations, establishing resident committees, and adding direct channels to sell biomass raw materials[60].”

**Response to Reviewer: 2’s Comments**

**Comment:** The overall logic of this paper is clear, and the game theory model is used to explore the optimal information sharing strategy for farmers to choose whether to occupy the biomass feedstock supply of biomass power plants. Through the detailed analysis of 8 different information sharing scenarios, a more powerful research conclusion is obtained. In addition, the structure of the paper is reasonable, and the selected research questions are interesting. For example, the research on the sustainable development of the biomass raw material supply chain is a very important research hotspot in China's current development and has strong research necessity. This paper chooses a good research entry point. Personally, I think it is a good research paper. However, there are still some shortcomings that need to be further improved to better meet the standards of public publication.

**Answer:** Thanks for your valuable comments and suggestions. We will answer your comments one by one.

**Comment (1):** Please adjust the expression of the first sentence of the second paragraph of the introduction. The second paragraph mainly expresses the view that "biomass power generation still has considerable space for development", which has no transitional relationship with the previous paragraph, it is not recommended to use "HOWEVER..." Sentence patterns to express ideas.

**Answer:** Thank you for your advice and we apologize for our error. We’ve changed the expression of the first sentence of the second paragraph of the introduction: “Although the Chinese government has been encouraging the development of biomass power generation in recent years, there is still considerable room for the development of biomass power generation.”

**Comment (2):** It is suggested to add "theoretical contributions" to the "6 Conclusions" section. This part should respond to the research gap proposed above through literature dialogue, in order to highlight the theoretical contribution of this research.

**Answer:** Thanks to your expert advice, we have added the following to Part 6: Contrasting the theoretical frameworks established in the current literature[61], our proposition posits that the excessive sharing of demand information by biomass power plants may paradoxically lead to negative outcomes, ultimately eroding their profitability. Biomass power plants must exercise caution in selecting their information sharing strategies, taking into account the intensity of competition within their respective supply channels. Consequently, our paper introduces a fresh perspective to the corpus of research exploring information sharing strategies in the context of biomass power supply, providing managers who must decide on information sharing strategies with practical insights and recommendations.

**Comment (3):** This research question has strong application orientation, and it is suggested to further enrich the content of "Managerial implications". Specific improvement measures can be proposed from the perspective of different relevant subjects, such as how the government should use the conclusions of this study to improve relevant work and so on.

**Answer:** Thank you very much. Based on your revisions, this paper organizes existing improvements from the perspectives of biomass power plants, intermediaries and farmers. The paper also adds three management recommendations using the government as a starting point. For your convenience, we repeat it here:

This will significantly improve the coordination and stability of the biomass power supply chain. Specifically, (1) Regardless of whether power plants share demand information, farmers should strive to expand their biomass sales channels as much as possible to enhance their own profits. (2) For middlemen, it is essential to actively pursue long-term cooperation with biomass power plants through various means in order to acquire demand information more rapidly and accurately, thus gaining a proactive position in the competition. (3) The demand information sharing strategy of biomass power plants can be affected by invasion scenarios. When farmers do not choose to invade, it is recommended that power plants share demand information exclusively with middlemen to ensure the stability of biomass feedstock supply; when there is an invasion, it is recommended that power plants share information with all upstream players as much as possible in order to broaden the feedstock collection channels. At the same time, when the competition intensity of the invasion channel is high, it is recommended that the power plants avoid sharing the demand information with the farmers exclusively, which may cause unnecessary price wars.

In addition to the specific members of the biomass power generation supply chain, this study also holds certain implications for government entities. As functional institutions, governments should strengthen the establishment of biomass information sharing platforms by publishing market information, collecting supply and demand data, and other means to provide support for decision-making among supply chain members. Furthermore, the government should formulate laws, regulations, and industry standards to regulate the order of the biomass market and promote fair competition. Additionally, the government can guide and encourage farmers to establish cooperatives for direct or indirect sales of biomass raw materials through incentive measures such as tax subsidies, thereby enhancing farmers' income and promoting the stable development of the biomass power generation supply chain.

We sincerely hope that you would be satisfied with the revised version of the paper. We would be looking forward to hearing any further comments and suggestions from you again.

Thank you very much!

Best wishes!
