# Supplementary material for: Information sharing and channel encroachment in biomass supply chains
Source: PLoS One. 2024 Sep 20;19(9):e0310539. doi: 10.1371/journal.pone.0310539 (PMC11414914; doi:10.1371/journal.pone.0310539)
Supplement: Attachment — Submitted filename: Response to Reviewers.docx [file pone.0310539.s003.docx]

**Authors response to** **Academic Editor's and Reviewers' Comments on** **PONE-D-24-05811R1.**

Dear Editor and Reviewers：

We would like to begin with our sincere appreciation for all the valuable comments, insightful suggestions and thoughtful corrections offered by the reviewers and editor to our manuscript (Information Sharing And Channel Encroachment In Biomass Supply Chains). The comments and suggestions definitely helped us to improve the quality of the manuscript. We have revised the manuscript in which major changes are highlighted in yellow. These changes are summarized below following a point-by-point response to reviewer's comments.

**Response to Academic Editor’s Comment**

**Comment:** Please review your reference list to ensure that it is complete and correct. If you have cited papers that have been retracted, please include the rationale for doing so in the manuscript text, or remove these references and replace them with relevant current references. Any changes to the reference list should be mentioned in the rebuttal letter that accompanies your revised manuscript. If you need to cite a retracted article, indicate the article’s retracted status in the References list and also include a citation and full reference for the retraction notice.

**Answer:** Thank you for your significant reminder. As per the stipulations, we conducted a thorough review of the references in the article, made revisions to certain references, and did not identify any articles in a retracted status.

**Response to Reviewer: 3’s Comments**

**Comment:** I now finished reading the manuscript of “Information Sharing And Channel Encroachment In Biomass Supply Chains”. This research the interactions between the information sharing strategy and channel encroachment in the straw power supply chain. I Think this paper is interesting, but several problems still need to be addressed.

**Answer:** Thank you very much for your kind comments on our manuscript. There is no doubt that these comments are valuable and very helpful for revising and improving our manuscript. In what follows, we would like to respond to the questions you raised and provide a detailed account of the changes made to the original manuscript.

**Comment (1):** In section3, there are two cases of whether farmers invade or not in d1, which should be further distinguished by using corner label. In addition, it is suggested that the decision sequence can be drawn in the form of a chart and then attached to explain, which is easy for readers to read and understand.

**Answer:** Thank you for pointing this out. We have relabeled all the requirements $d_{1}$in the paper and explained them in Table 2. Additionally, for the convenience of readers' comprehension, we have illustrated the decision sequence in a graphical format and provided explanations. For your convenience, we repeat it here:

(i). To better distinguish between the presence and absence of encroachment, we denote the demand in Channel 1 during encroachment as $d_{1}^{E}$, and the demand in Channel 1 without encroachment as $d_{1}^{N}$. The $d_{1}$ mentioned in the table and formulas has been updated.

(ii) As shown in Figure 2, the decision sequence is as follows: (1) Farmers decide whether to encroach; (2) After receiving the market signal f, the power plant decides whether to share information upstream; (3) The middleman first sets the unit price p_1_ for selling biomass to the power plant; (4) Farmers set the wholesale price w_1_ for selling biomass to the middleman and the unit price p_2_ for selling biomass directly to the power plant. This sequence illustrates the game relationships among farmers, middleman, and the power plant.

**Figure 2. Sequence of Events**

**Comment (2):** Table3 and 4 summarize the pricing conclusions in all cases, I suggest that the position should be adjusted, not in the case of 4.1Scenario NN.

**Answer:** We feel great thanks for your professional review work on our article. As you are concerned, we have adjusted the positions of Table 3 and Table 4 in the paper as follows:

(i). According to the article structure, sections 4.1-4.4 present the pricing equilibrium strategies for the four scenarios under the condition where farmers do not engage in incursion. Consequently, Table 3 has been strategically placed at the end of section 4.4 to succinctly summarize the equilibrium results for these four specific situations.

|  | NN | NF | NM | NFM |
| --- | --- | --- | --- | --- |
| $\boldsymbol{w}_{\mathbf{1}}$ | $\frac{1}{4}\left( a_{0}+3c_{1}-c_{2} \right)$ | $\frac{1}{4}\left( 4A-3a_{0}+3c_{1}-c_{2} \right)$ | $\frac{1}{4}\left( 3a_{0}-2a+3c_{1}-c_{2} \right)$ | $\frac{1}{4}\left( A+3c_{1}-c_{2} \right)$ |
| $\boldsymbol{p}_{\mathbf{1}}$ | $\frac{1}{4}\left( 3a_{0}+c_{1}+c_{2} \right)$ | $\frac{1}{4}\left( 3a_{0}+c_{1}+c_{2} \right)$ | $\frac{1}{4}\left( 2A+a_{0}+c_{1}+c_{2} \right)$ | $\frac{1}{4}\left( 3A+c_{1}+c_{2} \right)$ |

For convenience, the equilibrium pricing outcomes when farmers opt not to encroach the channels are presented in Table 3.

**Table 3. Equilibrium pricing for scenarios under the non-encroachment channel**

(ii) Similarly, since sections 4.5-4.8 depict the four scenarios where farmers choose to engage in channel incursion, we have placed Table 4 at the end of section 4.8 to summarize the equilibrium results for these four situations:

Table 4 shows the equilibrium prices under four scenarios for farmers choosing channel encroachment.

**Table 4. Equilibrium pricing by scenario in the encroachment channel**

|  | EN | EF | EM | EFM |
| --- | --- | --- | --- | --- |
| $\boldsymbol{w}_{\mathbf{1}}$ | $\frac{1}{4}(\frac{a_{0}(1+b)}{1-b}-bc_{0}+3c_{1}-c_{2})$ | $\frac{4A+a_{0}(-3+b)+(-1+b)(bc_{0}-3c_{1}+c_{2})}{4(1-b)}$ | $\frac{1}{4}(-2A+\frac{a_{0}(3-b)}{1-b}-bc_{0}+3c_{1}-c_{2})$ | $\frac{1}{4}(\frac{A(1+b)}{1-b}-bc_{0}+3c_{1}-c_{2})$ |
| $\boldsymbol{p}_{\mathbf{1}}$ | $\frac{1}{4}(\frac{a_{0}(3-b)}{1-b}+bc_{0}+c_{1}+c_{2})$ | $\frac{1}{4}(\frac{a_{0}(3-b)}{1-b}+bc_{0}+c_{1}+c_{2})$ | $\frac{1}{4}(2A+\frac{a_{0}(1+b)}{1-b}+bc_{0}+c_{1}+c_{2})$ | $\frac{1}{4}(\frac{A(3-b)}{1-b}+bc_{0}+c_{1}+c_{2})$ |
| $\boldsymbol{p}_{\mathbf{2}}$ | $\frac{1}{2}(\frac{a_{0}}{1-b}+c_{0})$ | $\frac{1}{2}(\frac{A}{1-b}+c_{0})$ | $\frac{1}{2}(\frac{a_{0}}{1-b}+c_{0})$ | $\frac{1}{2}(\frac{A}{1-b}+c_{0})$ |

**Comment (3):** The numerical analysis in Section 5. Will the change of the numerical value affect the conclusion? What are the values based on?

**Answer:** Thank you for your constructive comments. We have adjusted certain statements in Section 5，specifically citing references Wang et al. [50] and Liu et al.[56] to demonstrate the formulated range of values. The specific references are listed below:

[50] Wang T-Y, Li Y-L, Yang H-T, Chin K-S, Wang Z-Q. Information sharing strategies in a hybrid-format online retailing supply chain. International Journal of Production Research. 2021;59(10):3133-51.

[56] Liu P, Zhang R, Liu B. Information sharing under agency selling in an e-commerce supply chain with competing OEMs. Operational Research. 2023;23(3):1-27.

Similar to the two articles, the changes in these parameters may alter equilibrium pricing and profits, but they do not change the magnitude relationship between them. That is, they do not fundamentally affect our conclusion.

**Comment (4):** The analysis of proposition should also add the connection with reality, and put forward the corresponding management implications combined with the conclusion.

**Answer:** According to your nice suggestions, we have made corrections to our previous draft, the detailed corrections are listed below.

(i) In Proposition 2, We further analyze Proposition 2 from the perspective of farmers, considering real-world circumstances. “This conclusion aligns with reality. In practice, regardless of whether channel encroachment occurs, farmers should dismantle the information barriers inherent in traditional channels. They should proactively establish direct communication with biomass power plants via online platforms or on their own initiative. This approach ensures more effective information provision and thereby strengthens their decision-making process.”

(ii) In Proposition 3, We have added relevant analyses from the perspectives of middlemen and power plants. “In reality, middlemen should strengthen cooperation with biomass power plants and actively seek more accurate demand information to take advantage of the information gap and gain more benefits. Additionally, when there is an abundant supply of biomass raw materials, regardless of whether farmers conduct channel encroachment, power plants should give priority to providing middlemen with more accurate demand information to increase their own profits.”

(iii) In Proposition 4, We have analyzed the proposition from the perspective of biomass power plants, taking into account China's specific national conditions:“In practice, within China, the supply model for biomass feedstocks, which is predominantly controlled by middlemen, continues to maintain a substantial presence. To guarantee the stability of feedstock supply, biomass power plants must forge long-term cooperative alliances with middlemen, facilitated by the sharing of demand information.”

(iv) At the same time, we have revised certain expressions of Proposition 5 and Proposition 6 considering reality.

In Proposition 5:“This conclusion indicates that in a dual-channel structure, the power plant should actively forecast future demand and share demand information fully with upstream participants.”

In Proposition 6:“In reality, farmers should strive to broaden their sales channels for biomass feedstocks.”

(v) In 6.2 Managerial implications, according to your modification suggestions, this article combines reality to add relevant management inspirations for middlemen and farmers, and integrates them as follows: “Specifically, (1) From the perspective of farmers, regardless of whether power plants share demand information, farmers should strive to expand their biomass sales channels as much as possible to enhance their own profits. At the same time, actively seeking relevant information through both online and offline channels is a wise choice. (2) For middlemen, it is essential to actively pursue long-term cooperation with biomass power plants through various means in order to acquire demand information more rapidly and accurately, thus gaining a proactive position in the competition. In reality, middlemen should more appropriately stabilize their cooperation with power plants by adjusting their own pricing decisions, thereby reducing the intensity of channel competition.”

We hope you will find our revised manuscript acceptable for publication.

Yours sincerely!
